# Unilateral Ectopic Kidney in the Pelvis and Right Undescended Testicle: A Case Report

**DOI:** 10.7759/cureus.20095

**Published:** 2021-12-02

**Authors:** Zümrüt Doğan, Mine Farımaz, Yalçın Kırıcı

**Affiliations:** 1 Anatomy, Faculty of Medicine, Adiyaman University, Adıyaman, TUR; 2 Anatomy, Faculty of Medicine, Ufuk University, Ankara, TUR

**Keywords:** anatomy, vessels, cadaver, undescended testicle, pelvic kidney

## Abstract

Urinary system anomalies are cases that can be encountered in the clinic. In the anatomy laboratory, we observed that a different vessel was separated from the aortic bifurcation during routine dissection. As a result of the vascular follow-up, we determined that the male cadaver had a pelvic localized ectopic kidney. As a result of the vascularization, we found that there is an ectopic kidney with pelvic location in the male cadaver. One of the most common forms of congenital renal ectopia is pelvic localized ectopic kidney. When the dissection was detailed, the right undescended testicle accompanying the right ectopic kidney was detected. In conclusion we are of the opinion that knowing such anomalies and variations will guide physicians before surgical procedures.

## Introduction

The urinary system, which consists of kidneys, ureter, bladder, and urethra, forms the urine by filtering the blood [1]. Kidneys are organs located on the two sides of the vertebral column on the upper part of the posterior wall of the abdomen, lateral to the psoas major muscle [2]. Normally it is located on both sides of the abdominal aorta, just below the adrenal glands and its localization renal fossa [3]. Sometimes variations and anomalies can occur [4-6]. Congenital anomalies can take different forms. The most common form of congenital renal ectopia is the pelvic localized ectopic kidney [3]. The anatomical structure and variations of this region should be well known in the clinic.

In this case report, we describe a unilateral ectopic kidney with the right undescended testicle in a Caucasian male cadaver.

## Case presentation

Abdominal dissection was performed in a 69-year-old Caucasian male cadaver fixed by classical formaldehyde method in the Anatomy Department Application Laboratory of the Ufuk University Faculty of Medicine. We observed that a different vessel was separated from the aortic bifurcation during routine dissection. As a result of the vascular follow-up, we determined that the male cadaver had a pelvic localized ectopic kidney. When dissection was continued we determined that the right kidney was not in place. The left kidney is in normal position in the renal fossa; the right kidney was ectopic in pelvic location and smaller than the left kidney. The testicles were checked. The right undescended testicle accompanying the right ectopic kidney was detected in the variation.

**Figure 1:**
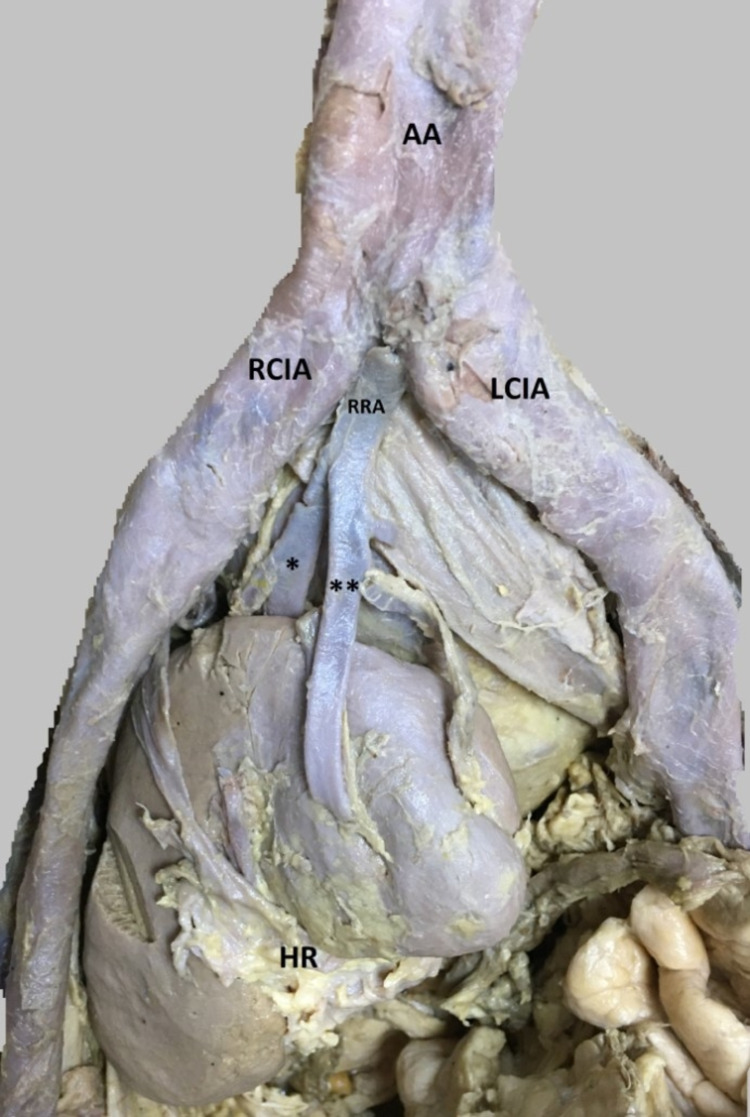
The arteries that feed the right kidney by separating from the anterior and posterior branches originating from the bifurcatio aorta AA: Abdominal Aorta; RCIA: Right Common Iliac Artery; LCIA: Left Common Iliac Artery; RRA: Right Renal Artery; HR: Hilum Renale *:  The posterior branch of renal artery feeding the right kidney; **: The anterior branch of renal artery feeding the right kidney

**Figure 2:**
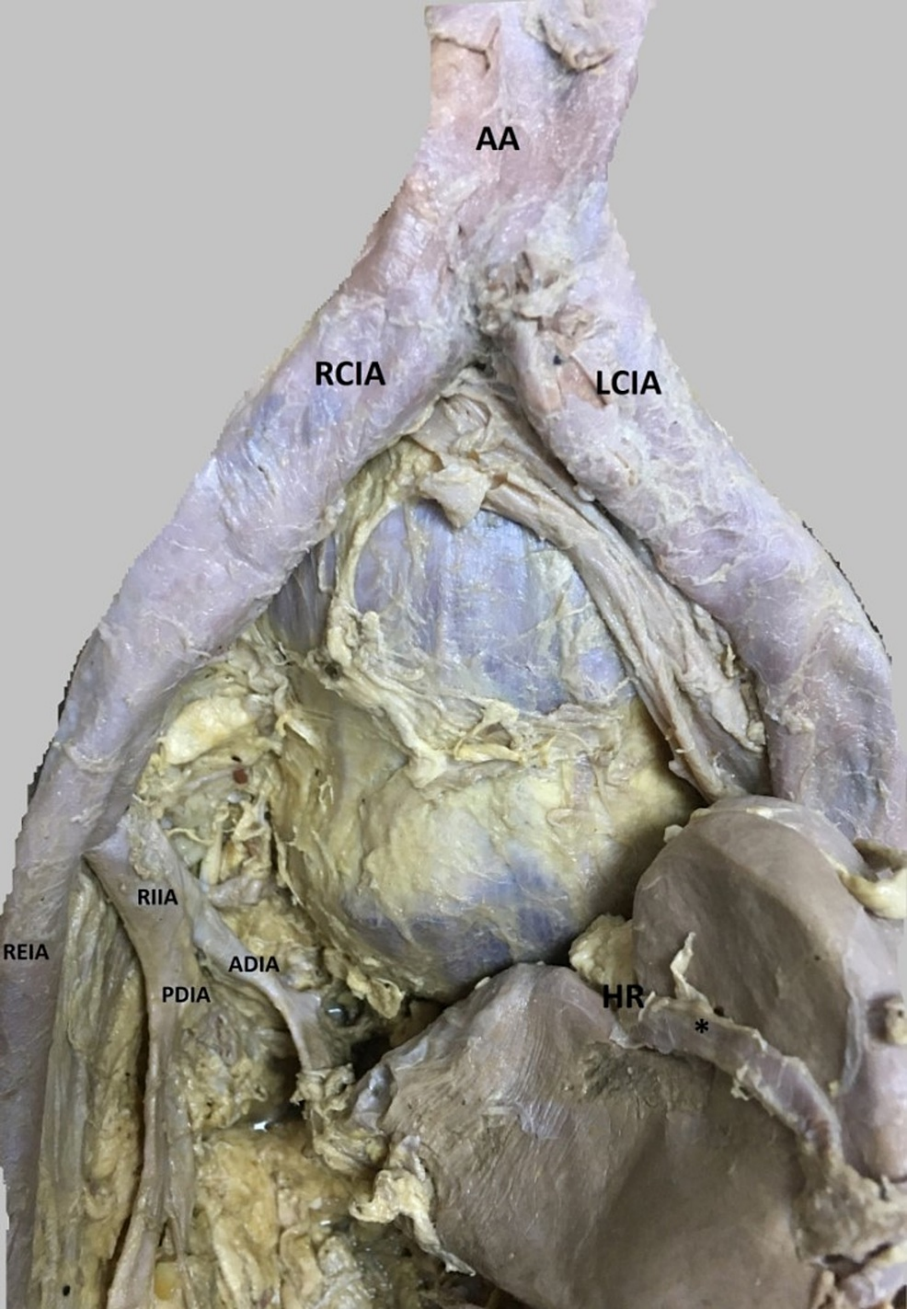
The artery feeding the extremitas inferior of the right kidney AA: Abdominal Aorta; RCIA: Right Common Iliac Artery; LCIA: Left Common Iliac Artery; REIA:  Right External Iliac Artery; RIIA: Right Internal Iliac Artery; PDIA:  Posterior Division of Internal Iliac Artery; ADIA: Anterior Division of Internal Iliac Artery; HR: Hilum Renale *:  The posterior branch of renal artery feeding the right kidney

In the 69-year-old Caucasian male cadaver, the abdominal region was dissected. The right kidney was located as an ectopic kidney with pelvic location (Figure 1). The left kidney is normally located 12x6x3 cm under the adrenal gland in the renal fossa. It weighed 150 g in size. The left renal artery emerged from the abdominal aorta at the L1-L2 level. It was seen that the left renal vein was poured into the inferior vena cava. The left renal artery was measured as 7 cm and the left renal vein as 13 cm. The right renal artery was 5 cm and the right renal artery come from the posterior division of the internal iliac artery was measured as 4 cm and the right renal vein was 11 cm (Figure 2). The left ureter was in its normal position and course.

**Figure 3:**
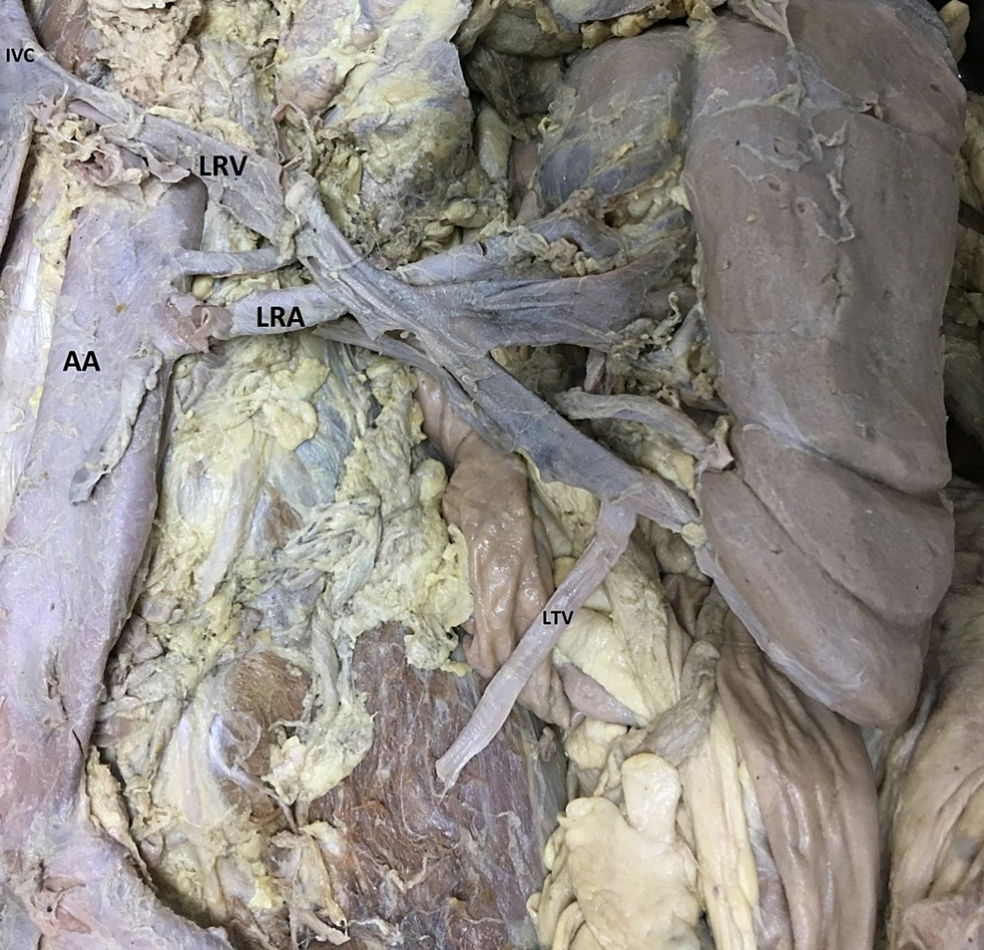
Vessels of the left kidney AA: Abdominal Aorta; IVC: Infeior Vena Cava; LRV: Left Renal Vein; LRA: Left Renal Artery; LTV: Left Testicular Vein

It was found that the blood supply of the left kidney was obtained from the aorta and the blood supply of the right kidney was obtained from the aortic bifurcation (Figure 1). It was observed that the renal artery feeding the left kidney emerged directly from the abdominal aorta and was 7 cm in length and entered the kidney from the renal hilum (Figure 3). It was seen that the abdominal aorta did not separate just below the superior mesenteric artery at the first and second lumbar vertebrae levels of the right renal artery. The right renal artery was not where it should be.

When the dissection is detailed, a branch emerging from the posterior trunk of the internal iliac artery to the inferior pole of the right kidney was determined. It was determined that this branch after giving the iliolumbar artery was separated from the lateral sacral artery and superior gluteal arteries branches (Figure 2). The other artery feeding the right kidney emerged from the anterior part of the aortic bifurcation as a thick root, dividing into two branches, one of which was in front of the right kidney and the other was on the back of the right kidney and had entered the renal hilum. The right ureter has entered directly from the body of the bladder. The right renal vein was opened to the common iliac vein. The right kidney's size and weight were measured smaller than the left. The right kidney was located in the space between the right internal iliac artery and the left internal iliac artery.

**Figure 4:**
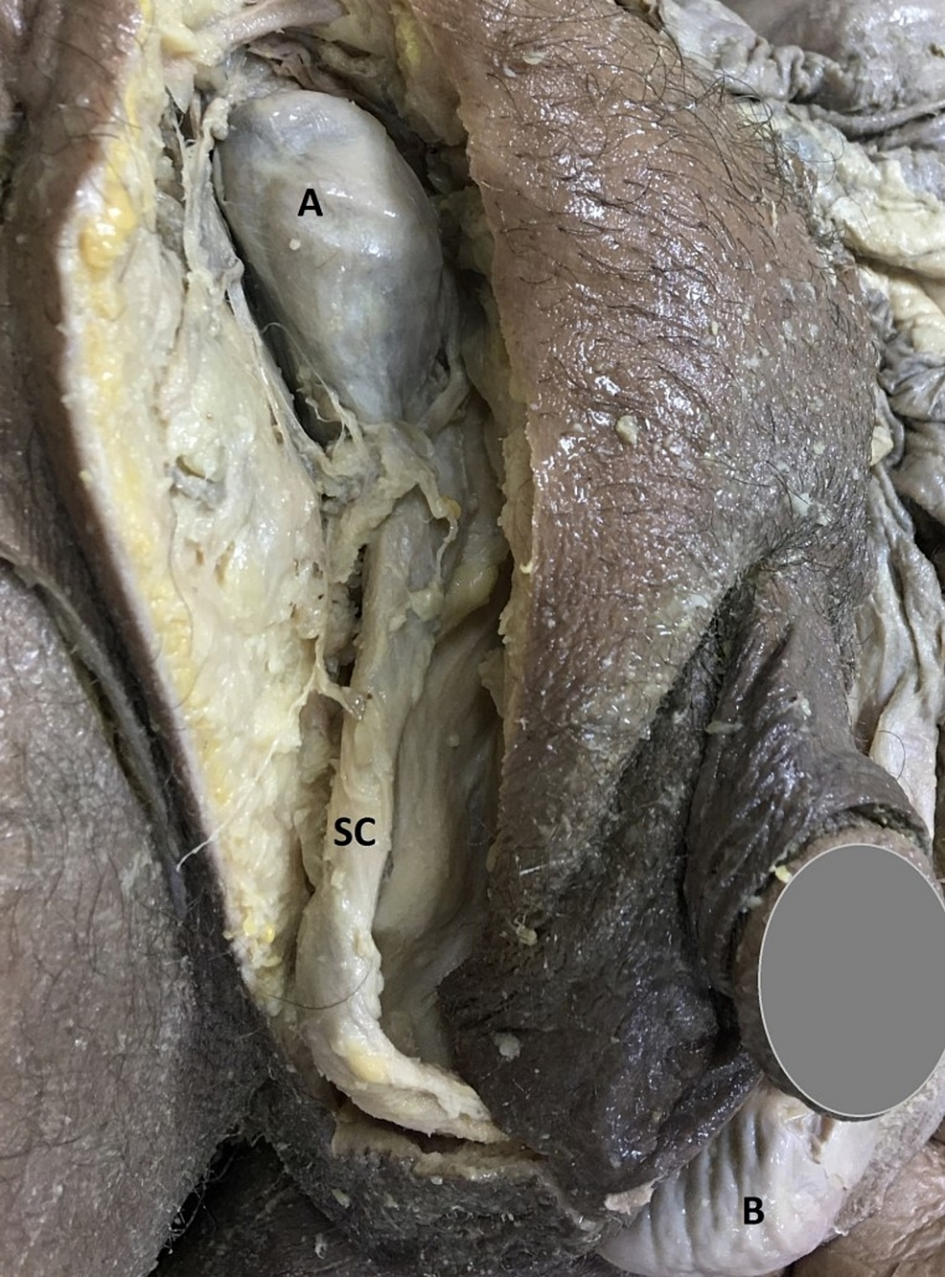
Undescended testicle in the cadaver A: Right testicle in the inguinal canal (undescended testicle); B: Left testicle in the scrotum (normal form); SC: Spermatic Cord.

When the scrotum was dissected, it was determined that the left testicle was in the normal position in the scrotum, and the right testicle was not in the scrotum (Figure 4). The right testicle was seen in the inguinal canal. Although the size and weight of both testicles were the same, the right undescended testicle was detected accompanying the right ectopic kidney. The right ureter was on the same side and entered the bladder.

## Discussion

Variations can be seen as a result of congenital anomalies in the kidneys, which are normally located in the fossa renalis on either side of the abdominal aorta. The most common congenital kidney anomaly has been reported as a horseshoe kidney [5]. Cross-ectopic kidney (CEU) anomaly is most common after horseshoe kidney anomaly. In the studies conducted, its incidence is given as 1/1000-7500 [6,7].

Liu et al. stated that CEU is more common in men than women and is generally on the right side [5]. Our case was also a male cadaver and supported this data as a right kidney ectopia. Simonds E and his team evaluated their cases as crossed fused renal ectopia (CFRE) since the renal artery was not in place and was located on the right side of the abdominal aorta [8]. In our case, the right renal artery was not in localization, it was fed from the bifurcatio aorta and internal iliac artery.

The fact that the right kidney is located in the pelvis, not in the renal fossa, and is smaller than the left kidney, supports the determination of ectopic kidney. In addition, the right ureter on the same side and entering the bladder also supports the fact. It has been reported in the literature that ectopic kidneys can be unilateral or bilateral [9]. In the light of these data, we defined our case as unilateral ectopic kidney.

Such anomalies are divided into six groups and are defined as sigmoid or S-shaped kidney, top kidney, L-shaped kidney, disc kidney, and superior ectopia of unilateral fusion kidney [3]. The right undescended testicle accompanying the ectopic kidney was also seen. We think that considering this data during surgical procedures will contribute to the clinic. To prevent problems that may occur during routine surgery, the positions of the ureters and vessels should be well known.

Soysal et al. reported a 38-year-old male patient with a pelvic right kidney. They stated that they did not have a right kidney in their upper abdominal ultrasonography. As a result of intravenous urography, it was determined that the patient had a right kidney with pelvic location. In CT angiography, the blood supply of the right kidney was observed from three different arteries, one from the abdominal aorta, one from common ıliac artery, and the other from internal iliac artery [4]. Such variations can sometimes be seen after differentiation during intrauterine development. In our case, we encountered during routine dissection, bifurcatio aorta of the right kidney, and It is seen that the internal iliac artery feeds on a branch originating from the posterior division. These data coincide with the findings of Soysal N [4]. We think that the existence of such anomalies should not be forgotten.

According to the data of Lossius et al., other anomalies can be observed with ectopic kidneys. Urinary and vaginal anomalies are seen accompanying the ectopic kidney in women; In men, cases such as undescended testicles, hypospadias, or urethral duplication have been reported [9]. In the case we dissected, there was a right undescended testicle. The right testicle was in the inguinal canal and there was no difference in weight with the left testicle. This variation data matches the literature [10].

## Conclusions

As a result, the ectopic kidney is a rare condition that is asymptomatic. We believe that the number of epidemiological studies on this issue is not sufficient. We think that our case report can contribute to the literature. In addition, we believe that anomalies are as important as the normal structure of the kidney. It is clear that determining such anatomical differences by various clinical methods and determining the procedure protocol accordingly will help the physician before the surgical procedures are planned.

## References

[1] Gray H, Standring S (2008). Gray's anatomy: the anatomical basis of clinical practice, 40th Ed. Gray's anatomy: the anatomical basis of clinical practice, 40th Ed.

[2] Snell RS (2007). Clinical anatomy by systems. https://books.google.co.in/books/about/Clinical_Anatomy_by_Systems.html?id=aZmUiAku3KMC&redir_esc=y.

[3] Dereli Y, Orhan A, Durgut K, Hoşgör K, Özdemir R (2014). Ectopic kidney giving appearance of iliac aneurysm [Article in Turkish]. Genel Tıp Dergisi.

[4] Soysal N, Koseoglu K, Sonmez HM, Karaman CZ (2005). Renal ectopy and renal artery anomaly in a young patient. Turkish J Nephrol.

[5] Simonds E, Iwanaga J, Kikuta S (2020). Case report of a pelvic crossed fused renal ectopic kidney. Kurume Med J.

[6] Wilmer HA (1938). Unilateral fused kidney: a report of five cases and a review of the literature. J Urol.

[7] AB BS, BH I (1959). Crossed renal ectopia with and without fusion. Urol Int.

[8] Liu DY, Wang HF, Xia WM, He HC, Shen ZJ (2015). Right-crossed, fused renal ectopia l-shaped kidney type with urinary chyluria. Urol Int.

[9] Lossius MN, Araya CE, Henry DD, Neiberger RE (2009). A patient with an unusual cause right lower quadrant pain and vomiting: pyelonephritis of an ectopic right kidney masquerading as acute appendicitis. Case Rep Med.

[10] Belsare S, Chimmalgi M, Vaidya S, Sant S (2002). Ectopic kidney and associated anomalies: a case report. J Anat Soc India.

